# Collagen I and the fibroblast: High protein expression requires a new paradigm of post-transcriptional, feedback regulation

**DOI:** 10.1016/j.bbrep.2015.07.007

**Published:** 2015-07-15

**Authors:** Richard I. Schwarz

**Affiliations:** Life Sciences Division, Lawrence Berkeley National Laboratory, Berkeley, CA 94720, United States

**Keywords:** PAT, primary avian tendon, ER, endoplasmic reticulum, P4-H, prolyl 4-hydroxylase, Collagen regulation, Feedback regulation, Post-transcriptional regulation, Prolyl 4-hydroxylase, Cell density signaling

## Abstract

**Background:**

Scaling protein production seems like a simple perturbation of transcriptional control. However, when embryonic tendon fibroblasts have to produce >50% procollagen and secrete it from the cell 4 times faster than the average protein, this taxes the cellular machinery and requires a fresh look at how the pathway is controlled. Ascorbate, a reducing agent, can stimulate procollagen production 6-fold. Procollagen mRNA levels goes up 6-fold but requires 3 days for the cell to accomplish this task. Secretion rates, the last cellular step in the process, also goes up 6-fold but this occurs in <1 h. What regulatory scheme is consistent with these properties?

**Scope of this review:**

This review focuses on fibroblasts that make high levels of procollagen (type I) and how they regulate the collagen pathway. Data from many different labs are relevant to this problem but it is hard to see the bigger picture from a large number of small studies. This review aims to consolidate this data into a coherent model and this requires solutions to some controversies and postulating potential mechanisms where the details are still missing.

**Major conclusions:**

In high collagen producing cells, the pathway is controlled by post-transcriptional regulation. This requires feedback control between secretion and translation rates that is based on the helical structure of the procollagen molecule and additional tissue-specific modifications.

**General significance:**

Transcriptional control does not scale well to high protein production with rapid regulation. New paradigms lead to better understanding of collagen diseases and tendon morphogenesis.

## Highlights

• Restricted to single copy genes, procollagen mRNA induction requires 3 days.
• Embryonic chick tendon cells use post-transcriptional, feedback control.
• Procollagen conformation allows feedback between secretion and translation rates.
• Prolyl 4-hydroxylase using its enzymatic and chaperone roles links these steps.
• Cell density controls enzyme activity and cell growth driving growth plate formation.

## 1 Introduction

Type I collagen is the most abundant protein in vertebrates being ~90% of the organic component of bones, tendons, and ligaments [1], [2]. This collagen forms fibers that are best known for their rope like structure with high tensile strength. Quantity is usually considered a simple variable, so little respect is given to cells for expressing a protein in large amounts. But high production puts stress on every part of the cell's machinery. If over half of the protein made by the cell is procollagen and the genes for the two chains are present in the genome as a single copy, what modifications are needed to make sufficient mRNA? If the cell secretes this protein, and the average cell secretes about 10% of its proteins, can the cell just speed up the assembly line and secrete 60–70%? Then there is the problem of control. Once the cell gets everything going at high speed, how does the cell rapidly slow production down and then speed it up again? In a tendon, for instance, the length of the tissue is critical for function and it is changing at every stage of development. A tendon that is too long or too short will not allow the muscle to accurately control the bone. A tendon that is too wide cannot act as spring and tendon that is too narrow will break. Therefore, controlling collagen production is critical in forming a functional collagen rope. So how do the regulatory mechanisms in high collagen producing cells differ from cells making relatively low levels?

Nature has supplied a highly specific inducer of collagen production, ascorbate (vitamin C, ascorbic acid). Ascorbate acts as a reducing agent, being especially suited to reducing ferric to ferrous [3]. This ability of ascorbate can increase procollagen production and secretion by 6-fold when cells are at a moderate to high cell density [4], [5]. In this review, nature's use of a reducing agent rather than a transcription factor to regulate type I collagen will be explained. This will require a focus on the feedback regulation between secretion rates and translation rates that depends on the helical structure of the collagen molecule. In turn, the activity of the enzyme, prolyl 4-hydroxylase (P4-H; EC 1.14.11.2), required to stabilize the triple helical conformation of collagen, will be shown to play a regulatory role. This enzyme uses molecular oxygen and ferrous ion to catalyze the hydroxylation of ~40% of the prolines in chicken collagen. Keeping ferrous ion reduced in the close proximity of molecular oxygen is the critical role played by ascorbate. In normal development in contrast to cell culture, where ascorbate levels are not limiting, the prolyl 4-hydroxylase activity levels become regulatory and the levels are controlled by cell density signaling. This signaling also controls cell proliferation and this causes tendon fibrils to grow by way of a cylindrical growth plate. This combination allows tendon cells to grow at the front of the growth plate, to make high levels of collagen in the middle, and apoptosis at the trailing edge. The consequence is that collagen uniformly fills a tube-like structure giving rise to a fibril that is the hallmark of this tissue. In the process a major irreversible transformation (in normal development) occurs whereby cell growth and differentiation occur on one side of the growth plate but on the other side, the few cells remaining are in a low growth, low collagen production, maintenance state.

In this review, we will fill in the details of the outline above and in doing so try to resolve some of the controversies. Some areas of the puzzle are only partially filled in and we will postulate solutions for how they may work.

## 2 Collagen regulation in high producing cells

### 2.1 Transcription

Regulating a pathway at the first step of protein biosynthesis, transcription, has a logical simplicity: the cell only makes what it needs and downstream controls are not required [6]. This common regulatory scheme could be used in regulating collagen in cells making lower levels of collagen. However, when avian tendon cells need to produce over 50% of their protein production as procollagen from single copy genes for the α_1_ and α_2_ chains, new problems are introduced. The amplification of a single copy gene by multiple copies of mRNA compensates for the limited rate at which a ribosome can translate a mRNA sequence into protein. The problem with high collagen production is that the cell needs 5000 times more mRNA than for the average protein. There is a limit to the rate at which the RNA polymerases can make mRNA off of a single copy gene (processivity). So if it takes minutes to fully induce an average protein, this process will take days to fully induce an abundant protein.

This is precisely what is observed in primary avian tendon (PAT, from 16 day embryos) cells. The induction kinetics can be done because nature has provided an almost specific inducer of collagen production, ascorbate. Surprisingly (since nobody knows why this should be true), chick tendon cells do not make ascorbate, despite the fact that they have the genes to do so. Instead, chick tendon cells rely on the liver or kidney to perform this task, and then secrete ascorbate into the blood, from where it is actively taken up [7]. As a result, with PAT cells at moderately high cell density (this requirement is discussed more below), one can add ascorbate and then follow the 6-fold induction of procollagen production. The procollagen mRNA goes up 6-fold but takes almost 3 days to linearly increase to the fully induced state (a 3-fold increase in transcription and a 2-fold increase in half-life [10.5–20 h]) [4], [8]. So regulation of collagen production by altering mRNA levels would be extremely sluggish for an embryonic chick tendon cell where type I collagen is first observed around embryonic day 10 [9] and the tendons have to be fully functional 11 days later.

### 2.2 Secretion

In stark contrast to transcription, ascorbate induction of procollagen secretion, the last cellular step in the pathway, is fast. In less than 60 min the rate increases 6-fold, and as a result, the half-life (the time it takes for half of the procollagen to leave the cell; a first-order reaction) drops from 120 to 20 min [10]. On the other hand, non-collagen proteins leave the cell with an average half-life of ~75 min and this is independent of ascorbate [10]. The average cell secretes about 10% of its total protein production and this rises to ~60% in PAT cells. The net result is that fully induced PAT cells make over half their total protein production as procollagen and secrete it almost 4 times faster than the average protein (~90% of the procollagen exits quickly but ~10% continues to take the slow route).

Ascorbate increases the hydroxylation of proline residues in the collagen part of the procollagen molecule to ~40% which is known to stabilize the collagen triple helix [11], [12], [13]. This occurs because hydroxylation favors pucker configurations of the proline rings that stabilize the trans conformation of the adjacent peptide bonds. This, in turn, is required for a stable collagen triple helix formation [14], [15]. The simplest interpretation of the kinetic data for collagen secretion is that a triple helical collagen has a faster route out of the cell than its non-helical counterpart [16], [17]. The kinetics also point to the transport out of the ER as the slow step in secretion. Because underhydroxylated procollagen can almost all be hydroxylated by activating P4-H with ascorbate, the pool of underhydroxylated collagen must be in the ER where P4-H resides [18], [19].

Other observations make procollagen secretion unusual. One relates to sensitivity of procollagen to endoglycosidase H. Procollagen I has N-linked glycosylations in the C-propeptide which when made in the ER can be cleaved by endoglycosidase H. However, these glycosylations are usually rearranged as the protein goes through the Golgi so that they become resistant to the enzyme. With collagen, both the cellular form and the secreted form remain sensitive to endoglycosidase H [20]. Another relates to the temperature insensitivity of the procollagen secretion process. The classic vesicle secretion model would predict that secretion would be very temperature sensitive – moving vesicles takes energy and these processes should slow with a drop in temperature. As expected with PAT cells, a 9 °C shift in temperature (41–32°; over this range secretion still follows first order kinetics) causes the secretion half-life of non-collagen proteins to increase 2-fold from 70 to 140 min. Procollagen secretion, both the fast and slow rates, remains stable over the same 9 °C change in temperature [21]. In summary, the above studies, from high collagen producing cells, have used kinetic and biochemical studies to probe procollagen secretion. They are consistent with two modes of exit from the cell, one of which is very fast, requires a triple helical collagen region, and the slowest step in the secretion pathway uses little energy; the other is slow, also the slowest step, exiting the ER, uses little energy, but does not discriminate on the helical nature of the collagen part of procollagen molecule.

**Fig. 1:**
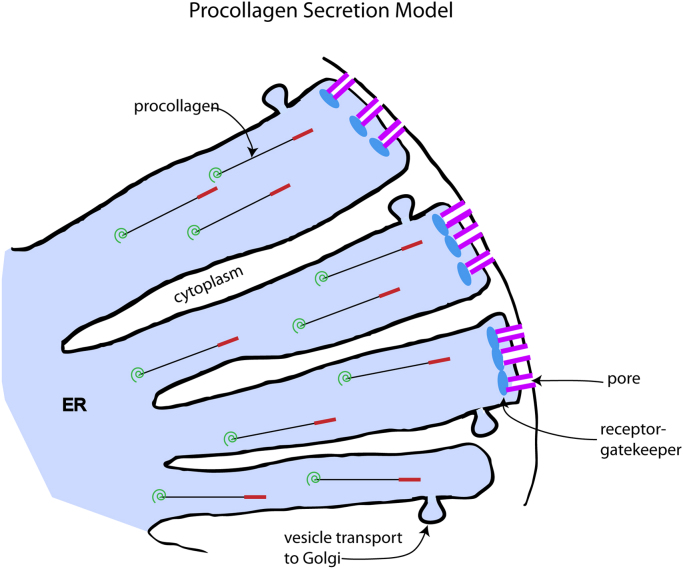
Hypothesis for a procollagen secretion model. The model for the fast route relies on the unusual characteristics of a triple helical procollagen molecule. The collagen triple helical region is a long and slender cylinder (300 nm×1.5 nm). The N-terminal propeptide (red) forms a hairpin loop and folds back and binds to the collagen helix. The C-terminal propeptide (green) crystal structure has been determined [46] but it is unclear whether it is flexible enough to slip through a small pore. We are suggesting that the procollagen molecule could be recognize by a receptor gatekeeper (blue; the slow step in the process) and go through a pore (purple) that transverses both the ER and plasma membranes. This would allow a fast exit from the cell that requires little energy, driven by a diffusion gradient. The slow route out of the cell is postulated to use vesicles. Packing large helical or non-helical stranded molecules into vesicles would be a slow process raising the half-life inside the cell by 6-fold. One can explain why ~10% of the triple helical procollagen goes our by the slow method in two ways. If there are many invaginations within the ER, some may only have the vesicle route. Alternatively, if the procollagen can take either route, then the ratio of vesicles to pores and the relative speeds of each step will determine the distribution that exits by each route.

These studies are in contrast to those using electron microscopy. The vast majority of these studies, but not all of them [22], conclude that collagen gets secreted using the classical method of vesicle transport from the ER to the Golgi and then vesicle transport to the plasma membrane [23], [24], [25]. The two techniques are very different: the kinetic studies reveal parameters but no details on the mechanism. The EM studies are a snapshot of a dynamic process and are biased towards those events that move slowly and are present in large amounts. Despite their differences, one can combine the data from both techniques and come up with a reasonable hypothesis for how procollagen secretion works in high collagen producing cells. The kinetic studies are consistent with two methods for procollagen secretion – slow and fast. The EM studies almost always see procollagen being secreted by vesicles through the Golgi. Electron microscopy studies are best at seeing slow steps, so we will assign the slow method to vesicle transport by way of the Golgi. However, kinetic studies have shown that the slowest step (the rate-limiting) is getting out of the ER. This slow step is assumed to be relatively temperature independent so it does not require lots of energy. In PAT cells we can put a time frame on this process: 120 min half-life to get out of the ER, potentially due to packing very long molecules into vesicles, then a relatively speedy trip to get to and through the Golgi and on to the plasma membrane (Fig. 1). Even when PAT cells are given ascorbate and all the procollagen is fully hydroxylated, ~10% still goes out by way of the slow route [10]. One can visualize this as a consequence of the ER having lots of invagination and roughly 10% only allow secretion by the slow process, or two competitive routes and vesicles are only 10% as efficient as the speedier pathway. Because of the high overall production, this is still a significant amount of procollagen going out through the Golgi and this is consistent with the electron microscopy observations. A similar argument would explain the observation that the secreted collagen's N-linked glycosylations are sensitive to endoglycosidase H. In this case, not all but only 90% remains sensitive to the enzyme and 10% is postulated to go out by way of the Golgi and become resistant to endoglycosidase H. This difference is hard to detect by protein gels unless one is really looking for it.

**Fig. 2:**
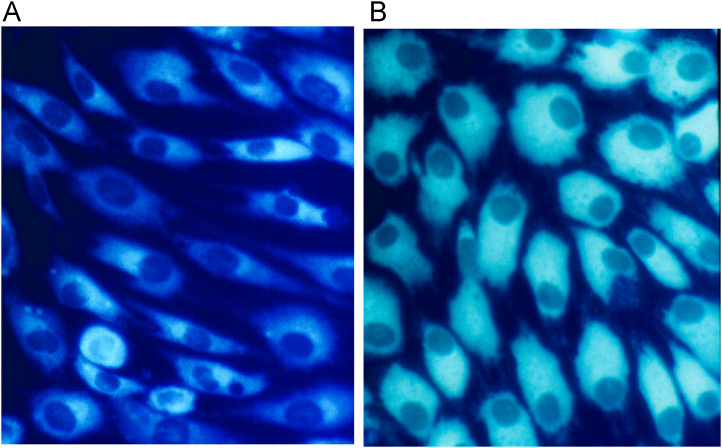
Ascorbate induction of collagen production in PAT cells causes a dramatic increase in the ER. PAT cells grown without ascorbate (A) and with ascorbate (B) were labeled with live cell dye highly selective for endoplasmic reticulum (ER-Tracker Blue–White DPX, Molecular Probes). As a consequence of the expansion of the ER in ascorbate induced cells, the nucleus is polarized to one side of the cell. Also, at this level of resolution, the ER appears to be very close to the plasma membrane. This expansion of ER could be important for secretion of procollagen and translation of procollagen mRNA.

The remaining question is how does procollagen leave the cell at the fast rate in high collagen producing cells. One observation may have relevance to this question. PAT cells show a dramatic change in cell shape when induced with ascorbate to make high levels of procollagen and this is related to the increased size of the ER. This increase in the ER is so great that it polarizes the nucleus to one end of the cell (Fig. 2). This also pushes the ER membrane in close proximity to the plasma membrane. One could postulate a pore crossing both membranes with a receptor gate keeper. Even though a triple helical procollagen molecule would have a high molecular weight (~450 kD), the helical part is contained in a long cylinder with a small diameter. The small diameter is important because a large pore would weaken the integrity of the membrane. Even the N-terminal propeptide forms a hairpin loop and folds back on the helical collagen [26], [27] and ensures that the leading edge retains a compact structure. The high concentration gradient would drive the process without the need for energetic steps. An alternative mechanism using secretory vesicles could be postulated whereby the vesicles go directly from the ER to the plasma membrane. With the expansion of the ER this would be a very short trip. As a consequence, it could go 6X faster and require less energy. This model would fit the kinetics but it is unclear how a vesicle would discriminate between a helical and non-helical conformations. In any case, the exact mechanism for fast secretion needs to be resolved.

### 2.3 Translation

After adding ascorbate, the first indication for increased translation occurs at ~3 h [4]. Stimulation of translation is intermediate between the fast change for secretion and the slow change for transcription. After a ~1 h delay to allow ascorbate to enter the cell, the pool of newly made procollagen would rapidly go from a non-helical to a helical form. This indicates that the two forms have the same degradation rates inside the cell since changes in degradation rates would be reflected in the translation rates. During ascorbate induction, translation rates are tethered to mRNA levels except that procollagen mRNA pool in the cytoplasm is used with twice the efficiency. This may reflect that when P4-H is in excess that this in itself stimulates translation by 2-fold As a result the 6-fold increase in protein expression is reached in 48 h at which point the efficiency tapers off as the P4-H levels again becomes rate-limiting. Translational control is revealed better by inhibiting collagen production in fully induced cells. Ascorbate cannot be removed from the cell by washing because ascorbate is actively taken up. Instead, P4-H can be inhibited by chelating ferrous ion with α,α′-dipyridyl [4], [17]. Adding the chelator to fully induced cells causes 2/3 reduction in procollagen translation relative to non-collagen proteins after 3–4 h. The uninduced cells show no effect of the chelator on the ratio of collagen to non-collagen production. However, chelating ferrous ion does have a non-specific effect on the cells and this causes all protein synthesis to drop by one third. Therefore on an absolute scale, one third of the drop in procollagen production is non-specific and two thirds is specific to procollagen. These results are consistent with a model where the cell is trying to keep the translation rates equal to the secretion rates. If the chelator causes a general reduction in protein synthesis, then the specific inhibition need only make up the difference to maintain the equilibrium between these steps. As a result, inhibition of collagen secretion causes a drop in collagen translation that restores the equilibrium between these steps in 3–4 h.

On the outside of the ER there is another important regulation involving membrane- bound ribosomes making type I procollagen composed of two α_1_ and one α_2_ chains. The genes are not linked, and are on different chromosomes in humans, so it is unlikely that the mRNA is made in a precise 2:1 ratio. In the case where the α_2_ chain is knocked out, and in some mutations, the α_1_ chain can form a stable trimer [28], [29], [30]. So what dictates the 2:1 ratio of these chains. One solution would be to have ribosome complexes with specificity for specific mRNAs. Distinct complexes of ribosomes have been observed in fibroblasts using electron microscopy [31]. There are reports of enhanced binding of procollagen ribosomes on the ER by a protein, p180 [32], [33]. A critical experiment was done where the DNA sequence for the promoter for type I collagen was inserted at the front of the type II collagen (cartilage) gene and then this construct was expressed in mice [34]. This directed cells in tissues that specifically expressed only type I collagen to now make large amounts of the type II procollagen mRNA. In well controlled experiments, those cells would not translate the inappropriate mRNA (<2% compared to collagen type 1 levels). This translational screening accounts for the lack of phenotypic change in those mice. While this result is striking, it makes sense that a cell would have to optimize several steps in a pathway in order to make high levels of a specific protein. That these optimized steps would have specificity for that protein is not completely unexpected.

### 2.4 Ascorbate, P4-H, and cell density

At this point ascorbate can be seen as regulating collagen production by keeping ferrous ion reduced, a requirement for the enzymatic activity of P4-H, and the resulting hydroxylation of prolines stabilizes the triple helical form of collagen. But there is a more dominant factor in this regulation: cell density. As was pointed out in the discussion above, ascorbate is only added to cells at a moderate to high cell density to induce the collagen pathway. At low cell density, adding ascorbate has no effect [5], [35]. Cell density turns out to be the key regulator of the collagen pathway and it works by controlling the level of one of the two subunits of P4-H [36]. So at low cell density the amount of enzyme becomes rate-limiting but at high cell density the active enzyme is ~6-fold higher and then, ascorbate levels becomes critical for maintaining enzyme activity. In normal chick development when ascorbate is always available, cell density through its regulation of active P4-H levels controls collagen production.

P4-H is an unusual enzyme. In vertebrates it is a tetramer (2α, 2β) [37]. The β-subunit is identical to a protein disulfide isomerase (rearranging disulfide bonds) that has multiple roles by itself, including being a protein chaperone. The complete enzyme has a high binding affinity for the single stranded form of collagen and a low affinity for the helical trimeric form [11], [13]. This differential binding always insures that the level of prolyl hydroxylation is sufficient to form a stable helix. In general the level of proline hydroxylation correlates with the denaturation temperature of the collagen produced [12]. However, the amino acid sequence of the collagen also influences helical stability [38].

The α-subunit is controlled by cell density. The ~6-fold increase in the α-subunit levels [36] as PAT cells grow from low to high cell density is not reflected in mRNA levels which remain relatively constant [39]. The cell density effect has been postulated to stabilize the protein through a change in the redox potential of the cell [39].

### 2.5 Hypothesis on translational control

The basic question is how does a high collagen producing cell coordinate the level of procollagen translation with its ability to secrete it? Or phrased from a developmental point of view, if the cell uses cell density to control P4-H levels how can this synchronize the rates of procollagen secretion and translation? Secretion is relatively simple. The cell has a slow and a fast method for secreting procollagen out of the ER. The more P4-H is active, the more procollagen becomes helical in the collagen region and can use the fast route for secretion. Translation is more complicated. Even physically, P4-H is inside the ER while translation is occurring on membrane-bound ribosomes on the outside of the membrane. The one thing that directly links these dominant players is the newly synthesized procollagen chains. Yet, changes in the activity of P4-H are sensed by the translational machinery.

**Fig. 3:**
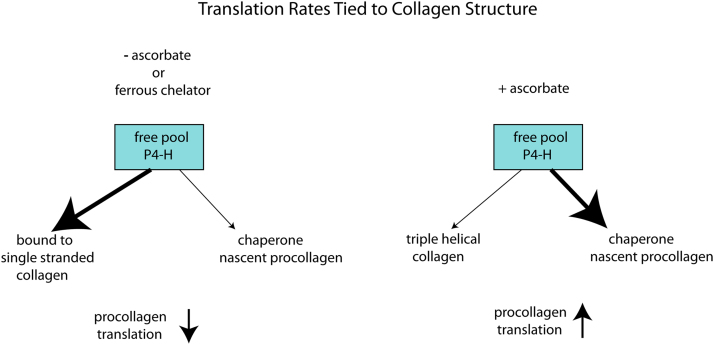
Translation rates are tied to the collagen structure within procollagen. We postulate that P4-H role as a chaperone to nascent procollagen is critical for rapid translation rates by reducing entanglements with other chains and proteins. Because P4-H has a low affinity for triple helical collagen, as soon as it has hydroxylated enough prolines to stabilize the helical structure it falls off and rejoins the free pool. Thus, for a given amount of P4-H an equilibrium is established allowing a certain rate of translation. If this is disrupted by a lack of ascorbate or use of a ferrous ion chelator, the P4-H remains attached to the single stranded collagen, and the free pool drops and as a consequence, translation slows down.

We postulate that the unique abilities of P4-H are designed for this task. First, P4-H has a strong affinity for the non-helical molecule and binds to it – a chaperone function. Partially due to the length of the procollagen molecule and the potential for the collagen portion to interact with itself, other newly formed chains, and other molecules, the role of chaperone could strongly impact the kinetics of translation. Second, when P4-H hydroxylates procollagen and the collagen region forms a triple helix, the enzyme loses binding strength and returns to the free pool. In this way the free pool level is linked to the activity of the enzyme and this in turn ties P4-H activity to the ability to act as a chaperone and stimulate translation rates (Fig. 3).

This model can explain the difference in lag time for secretion and translation induction. On addition of ascorbate there is an ~1 h delay for ascorbate to taken up and for P4-H to be activated. As the conformation of the procollagen in the pool changes, the helical procollagen gets secreted 6 times faster so it appears as a switch in rates. In contrast, the activity of the P4-H is not predicted to affect translation directly. As the triple helical part of procollagen is formed, the P4-H is released to the free pool where it can now act as a chaperone for newly synthesized procollagen. Building up the free pool can add another 30 m to the induction time. Then there is additional time needed to increase synthesis in order to significantly exceed the uninduced level. The net result is an ~3 h lag time.

While this model fits nicely with the known characteristics of P4-H and with the kinetics of induction, more research is needed to confirm its validity. Similarly, more research is needed to confirm or deny whether ribosomes are organized so that two α_1_ and one α_2_ chain can be produced in close proximity and whether hydroxylation and other modifications are occurring with active translation [40], [41]. Finally, more research is needed to understand the mechanism driving the expansion of the ER (Fig. 2) so there is room for more procollagen translation when PAT cells are fully induced.

### 2.6 Transcription revisited

As discussed above, transcription is the slowest step in the ascorbate induction process. There is a lag phase of 8–12 h and then a linear induction that takes ~3 days to increase the level of procollagen mRNA by 6-fold. The early lag phase seems inconsistent with induction by a transcription factor because these generally work on the order of minutes. Instead, one can speculate from what is seen inside the ER that pool size has a regulatory function. On the outside of the ER, as translation increases after 3 h, more of the procollagen mRNA is moving to membrane-bound ribosomes. This would cause a drop in the free pool in the cytoplasm that in turn could draw more mRNA from the nucleus that in turn could stimulate transcription. Such changes would be consistent with the slow induction but more experimental data are needed to clarify the induction of transcription and stabilization of procollagen mRNA.

## 3 Cell density and morphogenesis

**Fig. 4:**
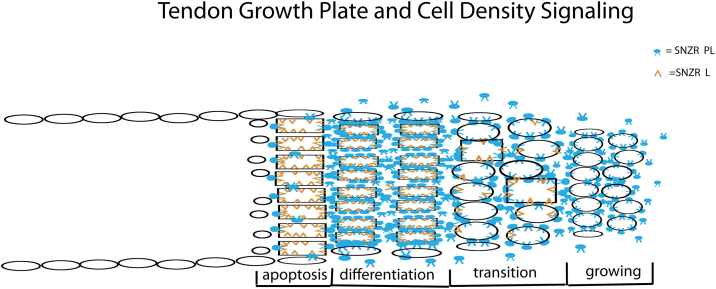
The tendon growth plate is regulated by cell density signaling (CC-BY 3.0 [45]). The signaling is composed of two components: a diffusible molecule (SNZR[sensor] PL, blue) that spends part of its time bound to the cell membrane is made up of a highly conserved peptide (P) bound to a tissue-specific lipid (L); a second molecule (SNZR L, yellow) which we postulate is the tissue-specific lipid is located in the plasma membrane. The amount of SNZR PL bound to the cell membrane reflects the level of cells in the vicinity. At a moderate cell density the level of SNZR PL is sufficient to fully stimulate proliferation. This level also stimulates the production of the free lipid. SNZR L. As SNZR L rises relative to the bound SNZR PL, the cell slows growth and raises collagen production allowing the growth plate to fill the “tube” with collagen (shown by oval cells becoming rectangles). At high levels of SNZR L, the production of the peptide slows. When the level of SNZR L is sufficiently high relative to SNZR PL, this triggers apoptosis – the cells are growing at the front and dying at the back allowing the growth plate to move. Basically, by having these two sensors, the tendon cell knows where it is, what it should be doing, and how long it has been doing it. All are essential for generating a growth plate. In the figure, at the bottom, are labels for the different regions of the growth plate. A few cells at the perimeter of the growth plate never reach a high cell density and escape apoptosis. These cells end up between the fibrils in a low growth, low collagen production, maintenance state. So the growth plate not only lays down a uniform collagen fibril but it causes a transition from a growing/differentiating state to a maintenance state.

From the discussion above, cell density is the major regulator of collagen production in tendon cells. Cell density is also the major regulator of cell proliferation. These are not new concepts. The ability of normal cells, as opposed to malignant cells, to limit their proliferation when they reach a certain cell density is seen both in vivo and in culture [42]. Similarly, the concept that cells have to slow their growth before they can differentiate is taken as almost axiom of cell biology [43]. But this regulation is taken to another level in chick tendon where these cells have to rapidly build a collagen rope [44]. Cells sense cell density by using a diffusible factor, a small protein bound to a tissue-specific lipid that spends part of its time bound to the membrane [45]. The higher the cell density the higher the concentration of the diffusible factor bound to the membrane. The consequence is that cell density regulation allows cells to form a growth plate [44], [45]. This was first predicted by mathematically modeling tendon morphogenesis using regulatory information obtained from experiments with PAT cells in culture and then confirmed by sectioning tendon tissue from the embryo and adolescent chicken at the middle of the tendon and at the muscle/tendon junction [21], [44]. The growth plate regulates collagen production in tube-like structure called a fibril (Fig. 4). The growth plate is also changing the state of the cells: the tendon cells in the growth plate are growing and differentiating but the few cells from the outer edge of the growth plate that escape apoptosis end up between the fibrils and are in a low growth, low collagen production, maintenance state. Therefore, cell density regulation of collagen production is part of a bigger morphogenic program.

## 4 Conclusions

High production of a single protein requires the cell to change its regulatory program. Transcription rates are restricted by a single copy gene and as a result building up large amounts of mRNA takes days. To overcome this, the cell accumulates large amounts of procollagen mRNA and then regulates the pathway at a downstream site.

High production of a secreted protein also requires high secretion rates. Vesicle secretion is slow as seen with non-collagen proteins and is sensitive to temperature changes. Instead tendon cells have a secretory route for procollagen with a helical collagen region that is 6X faster than the non-helical form. This route does not appear to go through the Golgi since secreted procollagen remains sensitive to endoglycosidase H and the secretion process is temperature independent over a 9° range. While we postulate two models, a pore through both the ER and plasma membrane with a receptor gate keeper, or vesicles that go directly between the ER and the plasma membrane, the exact mechanism is unknown.

To make a post-transcriptional regulatory step, one needs feedback between steps. Regulation is based on the amount of active P4-H. As the cells go from low to high cell density, there is a 6-fold increase in secretion and translation rates. To accomplish this, we postulate that translation rates rise with P4-H levels because of its ability to act as a chaperone and inhibit aggregation with itself, other procollagen chains, and other molecules. When P4-H activity is inhibited by ferrous ion chelators, the non-helical collagen never releases the P4-H for its role as a chaperone. Thus, the cell is using the structure of the collagen region to coordinate the regulation of both secretion and translation.

Because cell density signaling controls collagen production and cell proliferation, this signaling causes a growth plate to form that allows collagen to fill a tube with collagen and this is the building block of tendon morphogenesis.
